# Health-related quality of life in severe psychotic disorders during integrated care: 5-year course, prediction and treatment implications (ACCESS II)

**DOI:** 10.1186/s12955-022-02039-0

**Published:** 2022-09-08

**Authors:** Anja Christine Rohenkohl, Anne Daubmann, Jürgen Gallinat, Anne Karow, Vivien Kraft, Friederike Rühl, Daniel Schöttle, Martin Lambert, Romy Schröter

**Affiliations:** 1 Department of Psychiatry and Psychotherapy, University Medical Center Hamburg-Eppendorf (UKE), Martinistr. 52, 20246 Hamburg, Germany; 2 Department of Medical Biometry and Epidemiology, University Medical Center Hamburg-Eppendorf (UKE), Martinistr. 52, 20246 Hamburg, Germany

**Keywords:** Quality of life, Psychosis, Severe mental illness, Patient-reported outcome, Assertive community treatment, Integrated care

## Abstract

**Purpose:**

Studies on outcomes mapping Quality of Life (QoL) as patient-reported outcome over a longer period in severe psychotic disorders are scarce. However, such data would be particularly important for structuring, implementing and operating effective and efficient care models and for promoting satisfaction with care, service engagement and adherence.

**Methods:**

The ACCESS II study is a prospective long-term study of an integrated care model for people with severe psychotic disorders. The model includes Therapeutic Assertive Community Treatment within a cross-sectoral and interdisciplinary network. This publication analyses the course of QoL assessed with the Q-LES-Q-18 using a mixed model for repeated measures.

**Results:**

Mapping the course of QoL in *N* = 329 participants, there is a significant increase in the first 6 weeks of treatment (early course). Comparison to a published norm show significant lower QoL for severe psychotic disorders. The variable having a traumatic event before the age of 18 was significantly negatively associated with QoL. A decrease in the severity of depressive as well as in positive symptomatology in the first six weeks after admission was associated with increase of QoL.

**Conclusion:**

Results indicate that the overall symptom burden at time of inclusion is not decisive for the perceived QoL in the long-term course while the reduction in the severity of depressive and positive symptoms is important. This means focusing even more on the treatment of depressive symptoms and include traumatherapeutic aspects in the long-term treatment of severe psychotic disorders if needed.

***Trail registration*:**

ClinicalTrials.gov (identifier: NCT01888627).

## Background

Quality of Life (QoL) is a multidimensional construct that encompasses the functioning and subjective well-being in various important areas of life (including health). The World Health Organisation (WHO) emphasises that health is "a state of complete physical, mental and social well-being and not merely the absence of disease or infirmity" [1]. It is assumed that a person's QoL is influenced by the individual (health) situation and medical treatment. With the subjective reflection of health, the assessment of QoL has become one of the central patient-reported outcome (PRO) measures [2]. Due to the many advances in medicine from which people with a severe psychotic disorder also benefit through the further development of e.g. antipsychotic medication, long-term thinking in care is becoming increasingly important. Thus, outcome parameters are shifting and QoL is becoming more and more important as a relevant PRO to address satisfaction and well-being in a broader way than just being measurable by the presence or absence of (positive) symptoms [3–5].

As such, QoL has become an important issue in the care of people with (severe) mental disorders. Major reasons, beside the acceptance as an important criterion for treatment success, include the increasing community-based and patient-centered care, the importance of subjective well-being [6]. Although there is no universal definition of QoL, it is generally accepted that it contains both objective (e.g., mental and physical health) and subjective (e.g., feeling of well-being and satisfaction) dimensions [7]. Work on personal recovery (PR) also points to a subjective process in the course of (mental) diseases and incorporates various concepts such as connectedness, hope, identity, meaning in life, and empowerment (CHIME framework) [8, 9].Severe mental illness (SMI) is defined as the presence of a mental disorder, which cause severe episodic and/or chronic mental symptoms and thereby, severe impairment of social, personal, family and occupational functioning [7, 10]. This group of SMI thus also includes psychosis diagnoses, even in the early stages of the illness[11].

Patients with psychotic disorders, especially those diagnosed with schizophrenia exhibit an objectively severely reduced QoL at all stages of the disease. Compared to healthy controls, systematic reviews and meta-analyses have shown that patients in the early and late prodromal stages have a significant poorer QoL in all domains. This also applies to the early phase of the disease (early course) and in the long-term phase [12, 13]. The reduced QoL is expressed throughe.g. poor mental and physical health, problems in social relationships, and environmental domains such as living circumstances or finances [13]. Earlier results indicate the critical role of depression in determining QoL among early psychosis patients by demonstrating that greater baseline depressive symptom severity prospectively predicted poor QoL [14]. In addition, patients with psychotic disorders, who experience psychotraumatisation at an earlier age are known to report a poorer QoL compared with patients without a traumatic experience [15].

Evidence-based care including evident care models (e.g. Assertive Community Treatment (ACT)) and evident treatment components (e.g., pharmacotherapy, cognitive-behavioral therapy, social and somatic interventions) often led to an improvement in QoL [11, 16–18]. In view of the large number of risk factors for a poor course of the disease on the one hand and for discontinuous treatment on the other, special care models were developed for the early detection (Early Psychosis Services [16]), acute (Crisis Resolution Team [19]), as well as continuous (ACT) [20] treatment of patients with psychosis and specifically for those with SMI. However, studies on ACT as the only long-term care model for severe psychotic diseases show that patients treated with ACT often stabilize better than in standard care. In most cases they do not completely remit and still frequently interrupt or discontinue drug treatment as well as overall treatment [21].

Based on this overall situation, the improvement of patients' QoL is of particular importance. The QoL comprises a subjective measure of satisfaction with the individual circumstances of life and is, from a clinical point of view, an essential measure of the quality and success of treatment of patients and their relatives. Previous studies have often only targeted the defined cohort of first-time psychosis patients [22]. Results from long-term data in people with severe psychotic disorders are still scarce; whereby this would make a critical contribution for structuring, implementing and operating effective and efficient integrated care models and for promoting satisfaction with care, service engagement and adherence.

### Aims of the study

This study aims (I) to systematically assess the long-term course of self-reported QoL over a period of 5 years in a large sample of patients with severe psychotic disorders, fulfilling established criteria for SMI, (II) to compare data to a healthy norm and (III) to identify relevant variables influencing the course of QoL during the treatment period. A special focus will be placed on the early course of treatment, 6 weeks (42 days) after admission to the integrated care model, since we know that the lengths of stay (LoS) in psychiatric inpatient units varied from 17.9 days to 55.1 days in the European Union [23] and recruitment took place primarily through screening of inpatient admissions.

### Context

The ACCESS integrated care model for people with non-affective and affective severe psychotic disorders incorporates therapeutic assertive community treatment (TACT) within a multi-sectoral and interdisciplinary care network of inpatient and outpatient services from the adult and child and youth psychiatry of the University Medical Center Hamburg [11, 18, 24]. The effectiveness and efficiency of the ACCESS model was assessed within three studies: the ACCESS I study assessed the implementation of the model [25]; the ongoing ACCESS II study assesses all patients entering the model (approval by health insurances in Germany [18, 24]; the ACCESS III study evaluated the effectiveness of the expansion of the model to adolescents (from the age of 12 years) and young adult patients in the early stage of the illness [11]. In this evaluation, data from ACCESS II were used.

## Methods

### Study design and sample

The ACCESS II study is a prospective, single center, ongoing, long-term study assessing the effectiveness and efficiency of the *“Hamburg Model of Integrated Care (ACCESS)”* for people with severe psychotic disorders [11, 18, 25, 26]. Since this survey was implemented as quality assurance of the care concept, there is no control group. In the continuing ACCESS model, 471 patients have been treated to date in the period from May 2007 to June 2021. Patients were recruited for integrated care during inpatient stay via a weekly routine screening or in the acute phase of their illness by contacting the integrated care team themselves or via their psychiatrist in private practice to prevent inpatient stay. Those who participated in the program for at least five years and filled in the QoL self-report (*n* = 329; 75.98% of the total enrollment; Ø M = 9.78 yrs. (SD = 2.99)) were included in this analysis. The ACCESS trial was approved by the local ethics committee (number: PV4059) and is registered at ClinicalTrials.gov (identifier: NCT01888627).

### Inclusion and Exclusion Criteria

Inclusion criteria for the ACCESS II study were (i) age of 12 years or older, (ii) presence of one of the following psychosis diagnoses s according to the Diagnostic and Statistical Manual of Mental Disorders (DSM-IV-TR [27]: schizophrenia, schizophreniform disorder, schizoaffective disorder, delusional disorder, psychotic disorder not otherwise specified, bipolar disorder most recent severe with psychotic symptoms, and major depression, single or recurrent, severe with psychotic symptoms (summarized as severe psychotic disorder); (iii) written informed consent by the patient (≥ 18 years) or by guardians (parents or legal guardian) with written informed assent by patient (12–17 years) and (iv) a corresponding symptom load (Brief Psychiatric Rating Scale (BPRS) ≥ 40). Exclusion criteria comprised (i) presence of one of the following diagnoses according to DSM-IV-TR: Alcohol- or substance-induced psychosis (comorbid alcohol or substance abuse or dependence were tolerated), psychotic disorder due to a medical condition, and mental disability.

### Assessments and measures

Assessments were carried out at baseline (T1), week 6 (T2), and months 3 (T3), 6 (T4), and thereafter every 6 months (13 measurement times in total were included in this analysis) by trained raters. A special focus was placed on the first two measurement time points (early course: baseline (T1) and 6 weeks after admission (T2)), since we assume that the most changes occur at these time points with regard to psychopathology. All diagnoses were assessed as follows: (a) psychosis and comorbid mental disorders with the German version of Structured Interview I and, if indicated II for DSM-IV [28]; chronic somatic disorders and social support diagnoses (Z-diagnoses) with the ICD-10-GM [29]. Demographic and clinical characteristics (e.g. duration of untreated psychosis (DUP) were assessed with the Early Psychosis File Questionnaire (EPFQ [30], psychopathology with the Brief Psychiatric Rating Scale (BPRS; [31], functional level with the Global Assessment of Functioning Scale (GAF; [32] severity of illness for schizophrenia spectrum disorders with the Clinical Global Impressions Scale-Schizophrenia (CGI-S: CGI-S global, CGI-S cognitive, CGI-S positive, CGI-S negative, CGI-S depression [33]), childhood adversities scale adapted by Green, McLaughlin [34] and QoL with the Quality of Life Enjoyment and Satisfaction Questionnaire (Q-LES-Q-18) [35].

The Q-LES-Q-18 [35] is a validated self-report instrument assessing QoL for patients with schizophrenia, schizoaffective disorder, and mood disorder to cover their satisfaction with four specific life domains: physical health, subjective feelings, leisure time activities and social relationships. With these four domains, this questionnaire also includes the areas that are relevant according to the WHO definition, and grasp physical, mental and social aspects of well-being [1]. Each of its 18 items was rated on a 5-point Likert scale ranging from ‘not at all or never’ to ‘frequently or all the time’ depending on how often a person reports aspects of the QoL questions. Higher values indicate a better QoL. An index score is built as mean (SD) over all 18 items (total QoL score). Mean values were calculated when at least 80% of the items were completed. In order to better interpret and compare the values, the mean values were transformed to a value range from 0–100 with higher values being associated with a higher self-reported QoL.

### Statistical analyses

1. *Sample characteristics and course of QoL*: Sociodemographic data obtained from the general admission form at baseline (T1) were used to describe the sample characteristics. This was done through descriptive statistics by presenting frequencies and associated percentages for binary variables as well as means (*M*) and standard deviation (*SD*) for continuous variables. Means and standard deviation were also calculated to represent the baseline values of the clinical characteristics, later included in our analyses as independent variables, such as the BPRS, GAF, and values of the CGI-S scores as well as the dependent variables (Q-LES-Q-18 total QoL score and the four specific life domains).
2. *Comparison to a healthy norm*: To further describe our sample, a comparison with norm data of the Q-LES-Q-18 provided by Ritsner, Kurs [35] was conducted via a one sample t-test, reporting the test statistics T, *df* and *p* for significant results at baseline (T1) and after 5 years (T13). Norm data were obtained from a healthy norm population and later reported in the results section.
3. *Prediction of QoL over 5 years*: The course of QoL was analyzed with an explorative mixed model approach (Mixed Model for Repeated Measures; MMRM) for each, the total QoL score as well as the specific life domains: physical health, subjective feelings, leisure time activities and social relationships, assessed with the Q-LES-Q-18. Therefore, differences from baseline (T1) for each of the other 12 measurement points were calculated. As main effects the following variables were included, assessed at baseline (T1): age (continuous), gender (male or female), duration of untreated psychosis (DUP, continuous), type of diagnosis (affective versus non-affective psychosis), first- versus multiple episodes (dichotomous variable), comorbid psychiatric disorder (yes/ no), comorbid addictive disorder (without tobacco (yes/ no)), comorbid somatic disorder (yes/ no), positive family history with regard to psychiatric disorders (yes/ no), number of z-diagnoses (continuous), traumatic event (yes/ no), traumatic event before the age of 18 (yes/ no) as well as symptom burden (BPRS, continuous), assessment of functioning (GAF, continuous) and severity of the disease (CGI-S scores, categorical). To account for changes in clinical parameters in the early course, the differences from T2-T1 were included as independent variables additionally for the severity of the disease (CGI-S) and level of functioning (GAF). The burden of symptoms (BPRS) was only assessed every 6 months and is therefore not included in the variables used to display the early course. We evaluated changes over time considering the follow-up measures as repeated measures, the patients as random effect, time as fixed effects, the baseline values (T1) of the dependent variable (total QoL score and the four specific life domains) as covariate and controlled for age and gender. Outcome in this analysis was change from baseline in the total QoL score and the four specific life domains of the Q-LES-Q-18. When a main effect (independent variable) was not significant, the variable was successively removed from the model via backward selection. We used the baseline values as covariate to minimize the variance.

The main effects (*F*) and significance levels (*p*) were reported for significant results. For significant continuous variables, Pearson’s correlation coefficient (*r*) were reported to show the direction of the results. Level of significance was set at *p* < 0.05 (2-sided hypothesis). Due to the exploratory character of this study, we interpret the *p*-values in a descriptive manner and therefore do not correct for multiple testing. Statistical analyses were performed with SPSS Version 27.0 [36].

## Results

### Sample characteristics

471 patients were included in the ACCESS model from May 2007 to June 2021 in total, 337 completed at least a 5-year treatment period; of these, 329 participants completed the Q-LES-Q-18 as self-report at admission (T1) and were analyzed in this statistical evaluation. Sociodemographic and clinical characteristics at baseline of these 329 patients are displayed in Table 1. Concurrent with meeting the SMI criteria, all patients show high scores of psychopathology (BPRS: *M* (SD) = 78.32 (19.21)), severity of illness (CGI-S global: *M* (SD) = 5.62 (0.99)) and a low level of functioning (GAF: *M* (SD) = 38.48 (12.22)), assessed at baseline (time of admission). In addition, the number who answered “yes” to the question about trauma before the age of 18 is relatively high at 56%, even though the question was formulated very broadly and not specified in more detail.

**Table 1:** Clinical and sociodemographic characteristics

Clinical and sociodemographic characteristics at baseline				
		*N*	Mean	*SD*
Age (years)		329	36.70 (range: 14–80)	14.32
Gender	female	174 (52.9%)		
	male	155 (47.1%)		
*Clinical characteristics at Baseline (T1)*				
Type of psychosis	affective	92 (28.0%)		
	non-affective	237 (72.0%)		
No. of Episodes	first	92 (28.0%)		
	multiple	237 (72.0%)		
Comorbid psychiatric disorder	yes	262 (79.6%)		
	no	65 (19.8%)		
Comorbid somatic disorder	yes	200 (60.8%)		
	no	128 (38.9%)		
Comorbid addiction disorder (no tobacco)	yes	188 (57.1%)		
	no	139 (42.2%)		
Family history for mental illness	yes	196 (59.6%)		
	no	129 (39.2%)		
Traumatic event	yes	221 (67.2%)		
	no	106 (32.2%)		
Traumatic event (before age 18)	yes	183 (55.6%)		
	no	144 (43.8%)		
DUP …in weeks		253	63.64	92.60
No. of Z-Diagnoses		328	5.40	2.74
BPRS		325	78.32	19.21

Clinical characteristics early course	Baseline (T1)			6 weeks after admission (T2)		
	N	Mean	SD	N	Mean	SD
GAF	328	38.48	12.22	324	52.87	9.95
CGI-S global	329	5.62	0.99	327	4.52	0.85
CGI-S depression	329	4.14	1.42	327	3.49	1.09
CGI-S cognitive	329	4.13	1.35	327	3.22	1.0
CGI-S positive	329	4.86	1.68	327	3.37	1.33
CGI-S negative	329	4.01	1.48	327	3.44	1.21

Dependent variables (Q-LES-Q-18)	N	Mean	SD	N	Mean	SD
QoL total score	329	35.35	17.41	315	51.97	14.99
Physical health	329	33.24	18.53	315	48.15	16.76
Subjective feelings	328	37.77	21.20	315	56.01	18.03
Leisure time activities	327	32.74	22.96	315	53.28	18.41
Social relations	328	35.12	19.06	315	48.57	17.72

*BPRS* = *Brief Psychiatric Rating Scale; DUP* = *duration of untreated psychosis (weeks); CGI-S Score* = *Clinical Global Impression Scale (1–7); GAF* = *Global Assessment of Functioning Scale (0–100); Q-LES-Q-18 Scores are transformed from 0–100*

### Course of health-related QoL

**Fig. 1:**
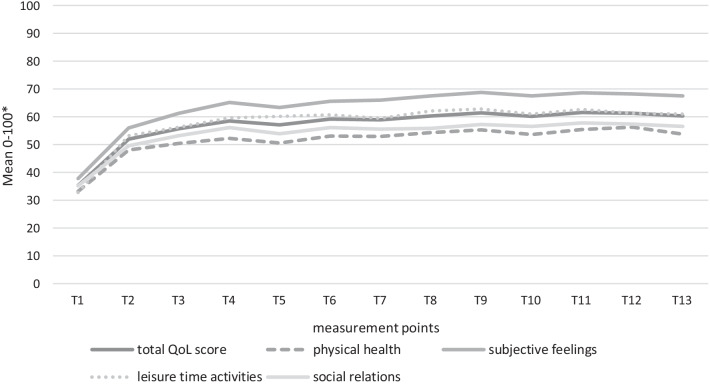
Five-year course in QoL. *Mean values of each scale were transformed to a range of values from 0–100; T1 = Baseline, T2 = 6 weeks after admission, T3 = 3 months after admission, T4 = 6 months after admission, and thereafter every 6 months

Figure 1 shows the overall course of QoL during the 5-year treatment period in the integrated care model (ACCESS II). There is an increase in self-perceived QoL in the early course of treatment in general which reaches a plateau after 3 months of treatment. Significant increases for the QoL total score between times of assessment can be reported from T1 to T2 (T = -5,535; *p* < 0.001) and from T2 to T3 (T = -2.19, *p* = 0.029). Later the mean values reach a plateau that maintains until the 5-year follow-up. Clinical relevant changes in self-reported QoL can only be seen in the early course after 6 weeks of treatment (95% confidence interval (CI) = -10.02 to -4.69; *M*_*difference*_ = 18.34, SD = 0.96). Equal results were found for the four specific life domains of the Q-LES-Q-18. As Fig. 1 shows, the self-perceived evaluation on the scale subjective feelings is the best with the highest mean values; whereas the evaluation on the scale social relations is the lowest. This result also includes that integrated care participants experience the most limitations in the QoL domain physical health.

### Comparison to a healthy norm

Compared to norm data reported by Ritsner, Kurs [35], this sample reported a significant lower total QoL at baseline (T1) as well as after a five-year treatment period (T13). This significant result is also found for all four specific life domains covered by the Q-LES-Q-18 (see Table 2).

**Table 2:** Comparison to a heathy norm

Q-LES-Q18 domains	Healthy norm	(T1) Baseline ACCESS		(T13) 5 years after admission to ACCESS	
	M (SD)	M (SD)	*T (df) p*	M (SD)	*T (df) p*
Physical health	4.1 (0.70)	2.33 (0.74)	− 43.32 (328) *p* < .001	3.15 (0.72)	− 17.11 (169) *p* < .001
Subjective feelings	4.4 (0.50)	2.51 (0.85)	− 40.35 (327) *p* < .001	3.70 (0.68)	− 13.42 (169) *p* < .001
Leisure time activities	4.0 (0.60)	2.31 (0.92)	− 33.27 (326) *p* < .001	3.44 (0.78)	− 9.41 (169) *p* < .001
Social relationships	4.1 (0.50)	2.40 (0.76)	− 40.26 (327) *p* < .001	3.26 (0.72)	− 15.19 (169) *p* < .001
Total QoL Score	4.2 (0.40)	2.41 (0.70)	− 43.92 (328) *p* < 001	3.41 (0.60)	− 14.88 (169) *p* < .001

### Prediction of Quality of life over 5 years

#### Total QoL score

After all sociodemographic and clinical variables listed in Table 1 being included in the explorative mixed model analysis, results show only a significant effect for the variable “traumatic event before the age of 18” (*F* = 8.71; *p* = 0.003). This group difference indicates that patients who reported a traumatic event before the age of 18, showed a lower QoL at admission to integrated care, but also rate their QoL lower over the 5-year period than patients who reported that there was no traumatic event before the age of 18. This result also implies that no other sociodemographic or clinical variable assessed at baseline has a significant impact on the 5-year course of QoL in severe psychotic disorders treated in the integrated care model (ACCESS II). The results described above show that QoL changes significantly, especially from T1 to T2. By including the changes in clinical parameters (differences in CGI-S scores and GAF from T1 to T2) as dependent variables in the mixed model, results show that the difference in the severity (CGI-S) for depressive symptoms (F = 16.47; *p* < 0.001) has a significant influence on the perceived course of QoL. The greater the decrease in CGI-S depression score within the first 6 weeks of treatment, the greater the gain in QoL (*r* = -0.246; *p* < 0.001).

The same MMRM approach was replicated for all four specific life domains separately, included as dependent variable in the models. In all domains the variable “traumatic event before the age of 18” as a significant effect (physical health (*F* = 4.35; *p* = 0.038), subjective feelings (*F* = 11.02; *p* = 0.001), leisure time activities (*F* = 12.16; *p* = 0.001) and social relations (*F* = 12.16; *p* = 0.001). Further deviating results will be reported for each domain/model below.

#### Physical health

The difference (T1-T2) in the severity (CGI-S) for depressive symptoms (F = 6.37; *p* < 0.012) as well as the CGI-S positive score at baseline (F = 6.37; *p* < 0.012) have a significant effect on the course of perceived physical health. The greater the difference from T1 to T2 in the CGI-S depression score, the more profit in the physical health domain and the higher the CGI-S positive score at baseline the greater is the increase in this domain.

#### Subjective feelings

For the outcome domain subjective feelings, the severity (CGI-S) global score at baseline has a significant effect (F = 4.22; *p* = 0.041); the more severe at baseline the more the gain in this domain. Results for this domain additionally provide significant results for the difference in CGI-S positive score (F = 6.25; *p* = 0.013); the greater the difference from T1 to T2, the greater the increase in subjective feelings. The CGI-S depression score at baseline (F = 4.06; *p* = 0.045) as well as the difference in CGI-S depression from T1 to T2 (F = 4.08; *p* = 0.045) have also a significant effect. The more severe at baseline in CGI-S depression, the greater the increase in the perception of subjective feelings in the 5 year course. The same is valid for the difference; the greater the difference in depression from T1 to T2, the greater the increase.

#### Leisure time activities and social relations

No further significant results can be reported for these domains.

## Discussion

In this analysis of the ACCESS II data, we aimed to shed light on the course of QoL in patients with severe psychotic disorders, treated with TACT as part of an integrated care model. So far, studies on outcomes that map the QoL as PRO over such a long period in severe psychotic disorders are very scarce. Even though the trend is moving more and more towards asking patients themselves about their QoL and thus also taking QoL into account as a primary outcome in studies.

### Key findings

As already shown in previous studies, the self-perceived QoL in severe psychotic disorders is lower compared to a healthy norm [12, 13] and thus is an expression of the overall severity of the diseases and associated everyday life limitations of this sample. This result is valid for the total QoL score as well as for all four specific life domains covered by the Q-LES-Q-18 assessed at baseline as well as 5 years after admission to the integrated care model, even if the mean values have continued to approach the norm in the course of treatment time.With these four domains, this questionnaire also includes the areas that are relevant according to the WHO definition and considered to be relevant with regard to subjective well-being, and grasp physical, mental and social aspects of well-being [1]. The course of QoL reaches a plateau after 3 months of treatment in integrated care (in QoL total score as well as in all subdomains); whereby the gain in QoL is greatest in the first 6 weeks after admission. The only sociodemographic variable that has a significant impact on the course of QoL over a 5-year treatment period was having or not having a traumatic event before age 18 (in QoL total score as well as in all domains). This was also shown in previous studies that focused on trauma in a sample with psychotic disorders [15]. Considering the clinical variables, the CGI-S global score at baseline has a significant effect on perceived subjective feelings. The CGI-S depression score at baseline has an additional effect on the physical health domain beside subjective feelings. Focusing on the early course of treatment, results show that a decrease in the severity of depressive symptomatology is crucial for a perceived increase in one's QoL (total QoL score, physical health and subjective feelings). To affirm depression as a critical determinant of prospective subjective QoL is also in line with earlier results [14]. A decrease in the severity of positive symptoms is relevant for an increase in the physical health domain and subjective feelings. Overall, the physical health domain as well as the subjective feelings domain of the Q-LES-Q18 were most sensitive and improved by clinical parameters. Significant results for the areas of leisure and social relationships could not be found, even though psychoses are linked to social isolation in the literature [37]. Compared to the norm, there are limitations, but we could not find a direct effect of clinical or socio-demographic variables in the long-term course. Here, it would be important to record the possible moderating effect of the care concept and, for example, to include participation in (therapeutic) groups.

### Limitations and outline

The ongoing ACCESS study assesses the long-term outcome in a sample of severe psychotic disorders. This study functions as an element of quality assurance of the integrated care concept and is not designed as a randomized controlled trial. Thus, there is no control group with which a comparison can take place over the course of 5 years and therefore, no generally applicable statements can be drawn from the present results. Results can be reported for this group with severe psychotic disorders treated in this integrated care model and remain observational. The present results lead us to ask, what exactly helps patients with a severe psychotic disorder? What elements in integrated care have a helpful impact on QoL? To date, there is no tool that covers this research question and examines the factors of effect in integrated care from the patient’s perspective. Studies on PR also show the importance of not only mapping the symptom course, especially in severe psychotic disorders. In addition to clinical and social factors, future research should also consider the interaction with subjective elements of PR [9]. Further data collection and adaptation of the ACCESS study should definitely address this.

Our data outline a relatively clear course with an increase in QoL in the first weeks and then the data remain on a plateau. Further analyses are necessary here to distinguish who, for example, arrives at a good QoL assessment in the course and who has a permanent limitation. A latent class analysis of this cohort would be necessary to answer this question.

For comparison with a healthy sample, we used the article published by Ritsner, Kurs [35] with 35 healty participants. However, the sample is small and no further sociodemographic data are available for the healthy group. A comparison with a healthy cohort similar to ours would certainly be useful and would have more validity in terms of information content. The question having or not having experienced a traumatic event before the age of 18yrs was not further specified and is therefore not subject to any generally applicable diagnostic criteria. Thus, a self-perceived trauma is to be assumed here.

In addition, current life situations and changes were not recorded. For example, the ongoing COVID-19 pandemic may also have an impact on self-reported QoL in severe psychotic disorders, which could be captured as an influencing variable in the future.

An explicit exclusion criterion in our ACCESS study and in our integraded care model was people without a permanent residence. Even though there is a significant proportion of people with a need for care, we did not include them. Finally, the ACCESS integrated care model has a special focus on the long-term treatment of severe psychotic patients but there are ways to extend the scope left. Further studies should, for example, also focus on the factors of effect in order to be able to show what patients find good, important and helpful about the model. Questions should go towards asking the patients themselves what is important in a care concept for more severe psychotic illnesses in order to increase or maintain the QoL.

## Conclusions

This analysis focused on QoL of patients a severe psychotic disorder while being continuously treated with TACT embedded in an integrated care system in a clinical routine setting.

The present results indicate that especially the first weeks of treatment are sensitive to subjective well-being. Patients’ self-perceived QoL benefits from a stronger reduction of depressive symptoms in die first 6 weeks of treatment. For clinical practice, this means that in addition to the treatment of positive symptoms in the acute phase, losses in mood should also be taken into account. This can be done, for example, through combined pharmacotherapy or increased use of psychotherapy with the provision of appropriate resources.

Through the present results, we know that experienced trauma before the age of 18 has a negative effect on the course of QoL. Thus, after acute phase and stabilization, it would be a possibility to include more trauma-specific aspects in the (psychotherapeutic) treatment and taking patients biography into account.

In summary, the results of the present study are promising, but to draw causal conclusions, stronger evidence including a long-term RCT focusing more on relevant impact factors on QoL would be required.

## Data Availability

The datasets used and/or analysed during the current study are available from the corresponding author on reasonable request.

## Abbreviations

ACT: Assertive community treatment
BPRS: Brief psychiatric rating scale
CGI-S: Clinical global impressions scale-schizophrenia
DUP: Duration of untreated psychosis
EPFQ: Early psychosis file questionnaire
GAF: Global assessment of functioning scale
LoS: Lengths of stay
M: Mean
MMRM: Mixed model for repeated measures
PRO: Patient-reported outcome
PR: Personal recovery
QoL: Quality of life
Q-LES-Q-18: Quality of life enjoyment and satisfaction questionnaire
SMI: Severe mental illness
SD: Standard deviation
TACT: Therapeutic assertive community treatment
WHO: World health organisation
